# Organo-Inorganic Hybrid Intumescent Fire Retardant Coatings for Thermoplastics Based on Poly(Vinylphosphonic Acid)

**DOI:** 10.3390/molecules25030688

**Published:** 2020-02-06

**Authors:** Baljinder K. Kandola, Katherine V. Williams, John R. Ebdon

**Affiliations:** 1 Institute for Materials Research and Innovation, University of Bolton, Deane Road, Bolton BL3 5AB, UK; kvwilliams91@gmail.com (K.V.W.); j.r.ebdon@btinternet.com (J.R.E.); 2 Exova Warringtonfire, Holmsfield Road, Warrington WA1 2DS, UK

**Keywords:** poly(vinylphosphonic acid), surface coatings, magnesium oxide, calcium silicate, chitosan, fire retardant

## Abstract

Thin coatings of crosslinked poly(vinylphosphonic acid), PVPA, display good adhesion and excellent intumescent, fire-retardant barrier properties when applied to the surfaces of a typical thermoplastic, such as poly(methyl methacrylate), but perform relatively poorly in water-soak tests. To strengthen and further improve the barrier properties of the intumescent char and to make the coating more hydrophobic, PVPA has been complexed with various inorganic and organic species. The chars formed from coatings of some of these hybrid materials are less friable than chars from coatings synthesized from crosslinked PVPA alone, and show higher levels of water tolerance with no significant reduction in dry adhesion to the substrate.

## 1. Introduction

Fire-protective and flame retardant coatings are a growing area of research offering, as they do, a convenient way of improving the fire resistance of pre-formed structures without requiring fire retardant additives to be incorporated or materials to be chemically modified at the manufacturing stage [1]. Coatings may be applied from solution, by electrodeposition, as powders or, as has more recently been demonstrated, by sol-gel and layer-by-layer assembly [2]. This last-named technique offers a convenient route to fire protecting complex substrates such as fabrics and foams [3,4]. Fire-protective coatings based on organic (usually polymeric) materials rely most often on the formation in a fire of an intumescent char. An intumescent char can, owing to its expanded porous structure, be particularly effective as a thermal barrier, retarding melting, and/or thermal decomposition and combustion of the underlying substrate [5].

Thin coatings (ca. 0.25–0.5 mm thick) of poly(vinylphosphonic acid) (PVPA) crosslinked with triallylisocyanurate (TAIC) have been shown to provide a very effective barrier to fire when applied to the surfaces of both thermoset resins, such as glass-fiber reinforced epoxy resin composites, and simple thermoplastics, such as poly(methyl methacrylate) sheet [6,7,8]. PVPA when exposed to fire, thermo-oxidatively degrades to give a highly intumescent carbon-rich char layer with a thickness up to 100 times greater than that of the original coating. This expanded char layer can substantially delay ignition (or even in some cases prevent ignition) of the underlying polymer and retard its combustion leading to much lower peak rates of heat release [7,8]. The intumescent char though is mechanically weak and, as is well known, the coherence of the char layer, along with its thickness and porosity, determine its thermal barrier efficiency [9,10]. While the char formed from a PVPA coating has adequate thickness and porosity, it lacks coherence and is mechanically relatively weak.

Another disadvantage of using crosslinked PVPA on its own as a fire-retardant (FR) coating is that although its adhesion to polymer substrates is generally strong, this adhesion becomes poor when the coated assembly is exposed to water for a significant length of time, e.g., in a 24 h water-soak test. It has been suggested that this loss of adhesion arises from swelling of the hydrophilic PVPA coating in water followed by accumulation of water at the polymer/PVPA interface leading to loss of attachment [8]. In a previous paper we reported experiments aimed at improving the water-resistance of PVPA coatings without detriment to dry adhesion and FR performance by chemically incorporating hydrophobic comonomer species, viz., greater levels of the hydrophobic crosslinker (triallylisocyanurate), diethylvinyl phosphonate, acrylonitrile, and dimethylsiloxane units [8]. For example, increasing the triallylcyanurate crosslinker content in the coating from 5 to 10 wt%, decreased the amount of coating removed from a poly(methyl methacrylate) (PMMA) substrate in an extended water soak test from 88% to 79%, whilst incorporating 20 wt% of acrylonitrile as a comonomer in the coating, reduced mass loss in a water-soak test to 47%, without significant detriment to dry cohesion or flame-retardant performance [8].

**Figure 1:**
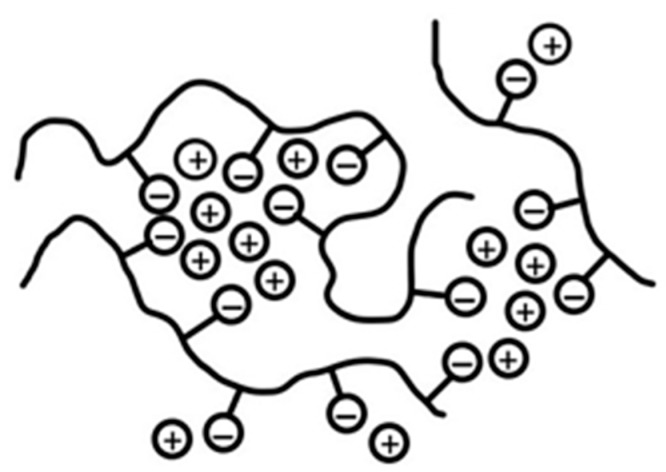
Schematic diagram of an ionomer showing crosslinks formed by ionic clusters in a polyanion with associated cations.

In this paper we report the incorporation into crosslinked PVPA of two inorganic species, namely magnesium oxide (MgO) and calcium silicate (CaSiO_3_), and the organic species, chitosan (a naturally occurring linear polysaccharide derived from the naturally occurring polymer chitin and composed of randomly distributed β-(1→4)-linked d-glucosamine and *N*-acetyl-d-glucosamine units. These additives were chosen in the belief that they might complex with PVPA to an extent sufficient to improve both the hydrophobicity of the PVPA coatings and also the coherence and mechanical strength of the chars formed from them. MgO was chosen owing to its reported inclusion with PVPA in some patented ionomer dental cement formulations [11] which are, by nature of their application, insoluble. CaSiO_3_ was selected with the expectation of forming water-insoluble calcium phosphate links in the PVPA (calcium phosphate is insoluble in water), thus improving water-tolerance, also owing to the possibility of silica contributing to the resilience of the char formed from the burning of a PVPA coating containing it. Silicates have been important constituents of many fire-retardant coatings formulations developed over the years [1]. In the metal salt/PVPA mixtures, it is expected that the polyvalent metal ions (M^2+^) will interact with the polyanionic PVPA to form ionic clusters that would act as cross links (see Figure 1) similar to those formed with other polyanions, such as polyacrylic and poly(styrenesulfonic acid)s [12].

Chitosan (a polycation) has been shown to be an effective FR for substrates such as cotton and polyurethane foams when combined in multilayer assemblies with polyanions such as clays, polyacrylic acid and phytic acid [13,14,15]. In our work with chitosan, PVPA is expected to act as a polyanion also.

## 2. Results

The characterization and properties of a crosslinked PVPA coating on a PMMA surface and as a disc (prepared by polymerizing the coating formulation on its own in an aluminum pan) have been discussed in detail in our previous publication [8] and are used here as a reference for new hybrid formulations.

### 2.1. PVPA/Inorganic Hybrid Coating

#### 2.1.1. PVPA/MgO Coatings

**Figure 2:**
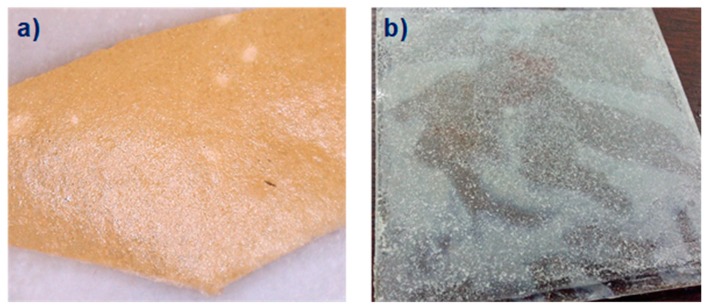
(**a**) Photograph of a PVPA/5% MgO coating showing regions of inhomogeneity (e.g., circled); (**b**) residual film of PVPA/1% MgO left on PMMA after a second water soak.

The MgO added to PVPA in these coatings is in a much smaller concentration (1%, 5%, and 10% wt%) than in dental cements. This was in order to maintain a relatively low viscosity, as formulations containing more than 10 wt% MgO were too viscous to be used as coatings. The coatings obtained were opaque, indicating that the MgO was probably dispersed in micro-crystalline (cubic, Halite-type) form, whilst at higher concentrations of MgO (5 and 10 wt%) it was clear that aggregation of MgO particles occurred, leading to visible heterogeneity of the coating as shown in Figure 2a).

**Figure 3:**
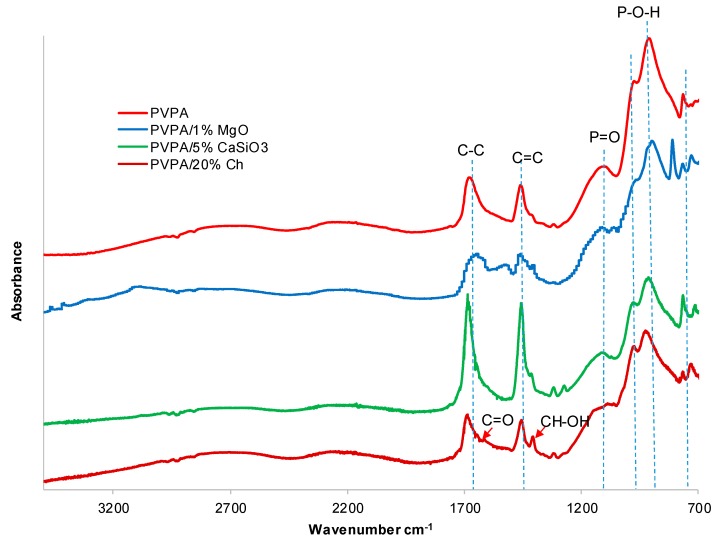
FTIR spectra of poly(vinylphosphonic acid) PVPA, PVPA/1% MgO, PVPA/5% CaSiO_3_, and PVPA/20% Chitosan (Ch).

**Figure 4:**
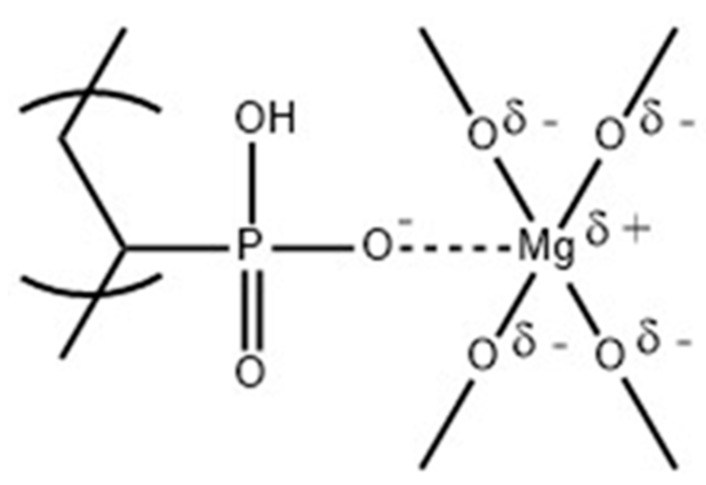
Proposed structure of PVPA/MgO complex.

The FTIR scans (Figure 3) of the coatings containing MgO did not show any marked difference from that of the unmodified PVPA coating. All the characteristics peaks of TAIC crosslinked PVPA, namely P-O-H stretches at 910 and 980 cm^−1^, P=O at 1100 cm^−1,^ C-H in plane bending at 1405 cm^−1^ and C=C stretching band (from incomplete consumption of all the C=C double bonds in the crosslinker) around 1640 cm^−1^ [7], are present. This is expected owing to there being no chemical reaction between PVPA and MgO, but merely the expectation of metal complex formation, most probably between phosphonate groups on PVPA and metal ions on the surfaces of the cubic microcrystalline particles of MgO, as shown in Figure 4.

The results of durability tests in the form of tape pull and water soak tests on the PVPA/MgO coatings when applied to PMMA plaques, and of water solubility tests on discs cast in aluminum dishes are given in Table 1.

The tape pull test results show that MgO-containing coatings have reasonable dry adhesion to the PMMA surface. As can be seen from the data in Table 1, when tested for adhesion to PMMA in the water-soak test, the coating containing 1 wt% MgO showed improved adhesion (54% mass loss) relative to that of PVPA alone (89% mass loss). The MgO containing coatings also left a residual film on the surface after soaking, which was not removed by a second water soak, as can be seen in Figure 2b).

**Figure 5:**
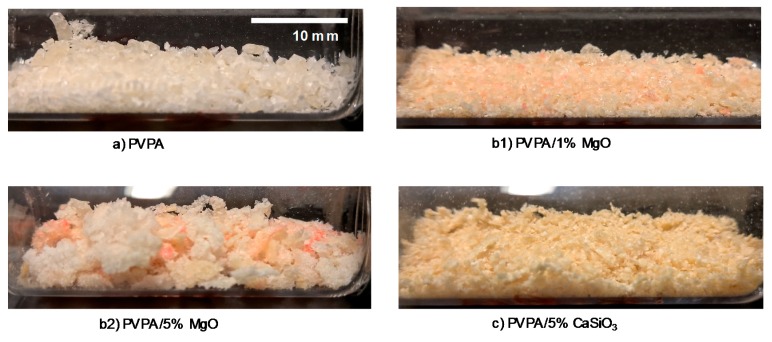
Images of post-water soak remnants of (**a**) PVPA, (**b1**) PVPA/1% MgO, (**b2**) PVPA/5% MgO and (**c**) PVPA/5% CaSiO_3_ discs.

In order to study the inherent solubility of the crosslinked coating in water, a pre weighed disc of each coating formulation was subjected to a water soak test. After the tests, undissolved disc residues were collected by filtration, dried, and weighed to assess the amounts that had dissolved. There is a slight increase in the water solubility of discs made from PVPA/MgO (18%, Table 1) compared with those made from PVPA alone (16%), which may be a consequence of MgO leading to a slight decrease in the extent of crosslinking of the PVPA. The residual insoluble flakes produced from a disc with 1 wt% MgO are smaller than those from PVPA alone, as can be seen from Figure 5(b1)), but the size increases with increasing concentration of MgO to 5 wt% (Figure 5(b2)), suggesting that the PVPA-MgO complexation improves hydrophobicity and improves the stability of the disc. The results in Table 1 also show that MgO helped to increase adhesion to the PMMA surface, possibly through complexation of the divalent metal ions of MgO with the ester groups of the PMMA (similar to the complexation observed between, for example, ester groups in poly(tetramethylene succinate) and various alkali metal salts [16]. However, the inherent solubility increased with increasing MgO (Table 1). This could be due to disruption of the crosslinking density of the PVPA by the MgO, particularly at higher concentrations of MgO. Also, at concentrations of MgO in PVPA of 5 wt% and above, there are visible signs of aggregation of MgO in the coating indicating that the films are heterogenous (Figure 2a), which may also contribute to their greater solubility. For this reason, we concentrated further on PVPA coatings containing only 1 wt% MgO.

**Figure 6:**
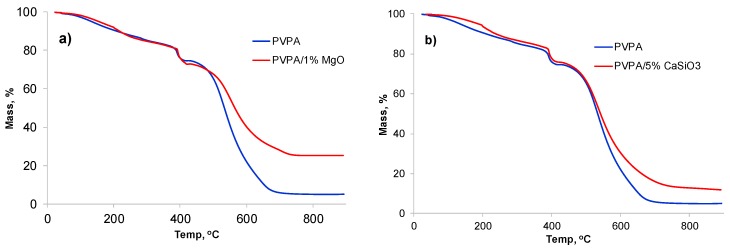
TGA curves under air of (**a**) PVPA, PVPA/1% MgO and (**b**) PVPA, PVPA/5% CaSiO_3._

A TGA trace of a 10 mg sample of the PVPA/MgO composite containing 1 wt% MgO recorded under air at 10 °C min^−1^ is compared with that of unmodified PVPA in Figure 6a). The TGA trace of PVPA shows that there are three stages of mass loss: 16.3% mass loss up to 340 °C, 9.1% mass loss between 340 and 422 °C and 69.4% mass loss between 422 and 750 °C. The first gradual stage of mass loss is where most of the water is released, and the second decomposition stage is where the polymer breaks down and the released water vapors help in intumescence (under fire conditions although not under slow TGA heating) The third stage from ~420–700 °C is where oxidation of char occurs. It can be seen that the introduction of 1 wt% MgO does not influence the first two decomposition stages, indicating that it does not interfere in the intumescence behavior. The mass loss rate in the third stage is low, leading to an increase in the amount of char at 800 °C of 25.1%. After subtracting the char yield of unmodified PVPA (5%) and the added percentage of MgO (1 wt%) the residual value of 19.1% shows that MgO promotes char formation in PVPA.

**Figure 7:**
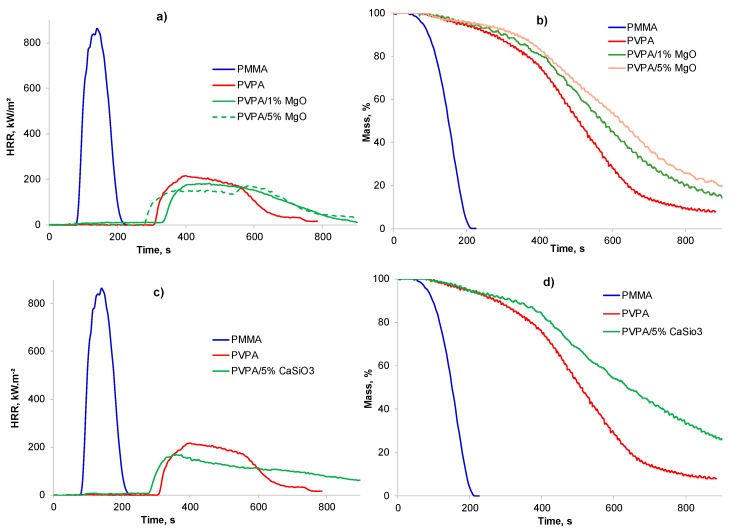
Plots of (**a**,**c**) heat release rate (HRR), and (**b**,**d**) mass loss vs. time of uncoated PMMA, PMMA coated with PVPA and (**a**,**b**) PVPA/1% MgO, (**c**,**d**) PVPA/5% CaSiO_3._

The burning behavior of a 75 mm × 75 mm plaque of PMMA coated with PVPA/MgO (1 and 5 wt%) was assessed by cone calorimetry at a heat flux of 35 kWm^−2^ with no source of ignition. The results of these tests are given in Table 2 and Figure 7.

PMMA, a highly flammable thermoplastic, ignites after about 89 s and burns with a high peak heat release rate (PHRR) of 900 kW m^−2^, and a total heat release (THR) of 78 MJ m^−2^, leaving no char. It can also be seen that a ca. 0.4 mm thick PVPA coating can prevent ignition of PMMA, although in replicate experiments it was found that ignition was not always prevented by such a thin coating. However, when ignition of PMMA does occur through the coating, the PHRR is considerably reduced and delayed, and the time to peak heat release (TTPHR) is increased. There is a little effect of the coating thickness on reduction of PHRR and THR from that of PMMA (see Table 2), however char height with no ignition was greater (32 mm) compared to when it ignited (~22 mm, Table 2). The mechanism of intumescence of PVPA coating has been discussed in detail elsewhere [7,8].

**Figure 8:**
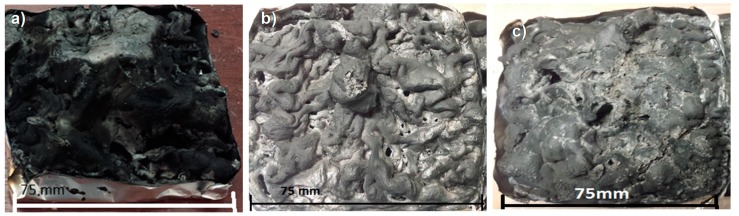
Photographic comparison of intumescent chars formed after cone exposure from (**a**) PVPA, (**b**) PVPA/1% MgO, and (**c**) PVPA/5% CaSiO_3_ coatings on PMMA.

It is clear from the results in Figure 7a and Table 2 that PVPA/1% MgO coatings offer fire protection to PMMA substrates similar to that offered by PVPA alone with complete prevention of ignition or delayed ignition and greatly reduced rates of weight loss. There is no advantage in increasing the MgO concentration to >1%, as even 1% MgO provides efficient fire protection. While there is a little difference in the intumescence behavior in terms of char thickness, char formed from PVPA/1% MgO is more rigid and less friable. The morphology of the char from PVPA/MgO is very different from that from PVPA: It is of a lighter grey color, suggesting that the white MgO is dispersed throughout the char, (see Figure 8b), and has a worm like structure.

#### 2.1.2. PVPA/CaSiO_3_ Coatings

The visual appearance of the coating containing 5 wt% CaSiO_3_ was similar to that containing MgO, i.e., reasonably uniform coatings on PMMA with minimal aggregation of the CaSiO_3_. Concentrations of CaSiO_3_ higher than 5 wt% led to aggregation. The IR spectrum was similar to that of PVPA/1% MgO, with no new absorbances evident.

In a tape-pull test only 0.17% of the coating was lost (Table 1), indicating strong adhesion to the PMMA surface. This suggests that the calcium silicate has not interfered with any bonding occurring between the PVPA and the PMMA. However, in the water-soak test, 97% of the coating was removed. During an inherent solubility test of the disc, the coating disintegrated completely, with the insoluble flakes being much smaller and less distinct than those from unmodified PVPA (see Figure 5c). The total mass retained at the end of water soak testing was 85.3%. This is most likely due to the small, but finite, solubility of calcium silicate in water (about 0.01%, compared to the total insolubility of MgO in water), which would therefore not significantly inhibit swelling and osmotic rupture of the coating. It is clear from these results, therefore, that PVPA/CaSiO_3_ performs less well as a mechanically and water-resistant coating over PMMA than does PVPA alone.

A TGA run on PVPA/5% CaSiO_3_ in air shows that addition of CaSiO_3_, while not adversely affecting the first and second stages of mass loss, leads to only a marginal increase in char yield of about 3 wt%, once the amount of added CaSiO_3_ is subtracted from the final mass (13 – 5 = 8 wt% for PVPA/5% CaSiO_3_ vs. 5 wt% for PVPA). This suggests that, unlike MgO, CaSiO_3_ does not interact strongly with PVPA such as to contribute to char formation but acts essentially as an inert, fire-resistant filler. This however, is not expected to interfere with the intumescence properties of the PVPA coating. As indicated above, overall the PVPA/5% CaSiO_3_ coating performs much less well than the PVPA/1% MgO coating in the water-soak test (97% removed vs. 54% removed) and even worse than PVPA alone (89% removed) suggesting that CaSiO_3_ has an anti-adhesive effect on the bond between PVPA and PMMA. The fire-protective effect of PVPA/5% CaSiO_3_ was, however, comparable with that of PVPA (see cone calorimetric traces in Figure 7c).

The char produced from the combustion of PVPA/5% CaSiO_3_ was similar to that from PVPA/1% MgO, i.e., with an intumescent cellular structure, slightly greyish in appearance, mechanically fairly robust, and with few major voids (Figure 8c).

### 2.2. PVPA/Chitosan Coatings

**Figure 9:**
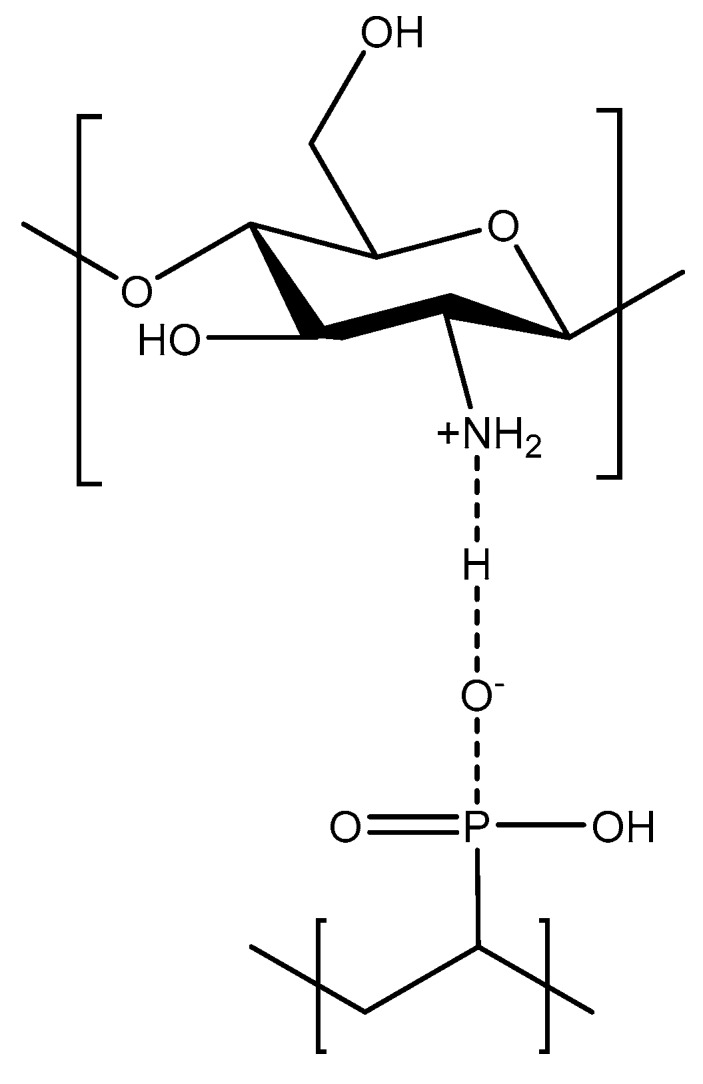
Schematic indicating acid-base pairing between chitosan and VPA monomer units.

Chitosan was chosen to include within a PVPA-based coating with a view to improving both hydrophobicity, as chitosan is soluble only in acidic media, and fire retardance, as it contains nitrogen-containing amine groups, which might be expected to act synergistically in this respect with the phosphorus in PVPA. PVPA and chitosan are expected also to interact via acid–base pairing (Figure 9). PVPA coatings containing chitosan were prepared (i) by a sol-gel type process [17] and (ii) by simply mixing small amounts of chitosan with vinylphosphonic acid (VPA) prior to polymerization. The results of tape-pull, water-soak and water-solubility tests on these coatings on PMMA and of discs made from them are given in Table 1.

**Figure 10:**
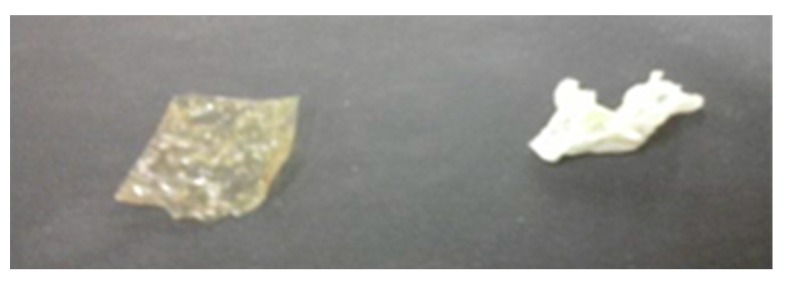
PVPA-Chitosan sol gel films before and after water soak testing.

As can be seen from Table 1, the disc prepared from the PVPA-chitosan gel proved to be relatively insoluble in water with only 37% of the coating dissolved following soaking in water for 24 h and with the disc remaining relatively intact, as seen in Figure 10. This shows that complexation with chitosan would improve the hydrophobicity of a PVPA based coating. However, when this gel was applied as a coating to a PMMA plaque there was very poor adhesion to the surface of the PMMA, with 96% of the coating removed in a tape-pull test. This formulation is therefore unsuitable for use as a coating and no fire tests were therefore carried out on it. It is possible that this reduced adhesion arises from dilution of the strongly adhering PVPA with less adhesive chitosan at the interface between the coating and the PMMA.

However by mixing small amounts of chitosan with the VPA/TAIC/HMD polymerization mixture prior to UV-photopolymerization (method (ii)), satisfactory coatings could be spread on the PMMA surface and discs could be made. The FTIR spectrum of PVPA/10% chitosan, shown in Figure 3, exhibits the same key characteristic PVPA peaks, such as P-OH at around 910 and 980 cm^−1^ and P=O at 1100 cm^−1^, along with absorbances characteristic of chitosan at around 1660 cm^−1^ (C=O), and 1420 cm^−1^ (CH-OH) [18].

**Figure 11:**
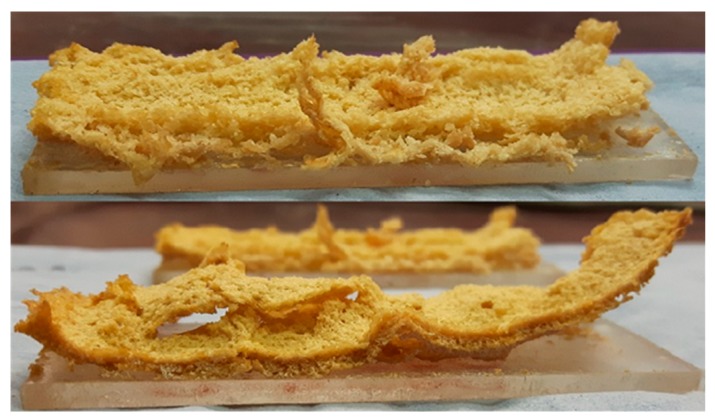
Coatings of PVPA/10% chitosan (**top**) and PVPA/20% chitosan (**bottom**) applied to PMMA after water soak testing.

The coatings applied to PMMA plaques showed the best combination of dry and wet adhesion performance in tape pull and water soak tests, especially with chitosan at a concentration of 10 wt% (see Table 1). Although the tape-pull test performance of the PVPA/10% chitosan coating is inferior to that of PVPA alone (0.67% removal vs. 0.04% removal), it is still acceptable, and its performance in the water-soak test is considerably better (24% loss after 24 h vs. 89% loss). The slight reduction in adhesion between the coating and the surface of the PMMA is due possibly to the chitosan molecules adhering less strongly to the PMMA surface and thus reducing the high a degree of bonding between the PVPA and the PMMA as is observed in samples without chitosan. The decrease in mass loss during water soak testing on the coating applied to PMMA is possibly due to the hydrophobicity of chitosan creating a protective effect for the PVPA. Despite the increase in the mass retention on PMMA, the coating does appear to be peeling away from the surface of the substrate after water soak testing, most notably in the PVPA/20% chitosan sample. This is shown in Figure 11. The inherent solubility of the disc though was same as that of PVPA, the disc remained intact, similar to that shown in Figure 11.

**Figure 12:**
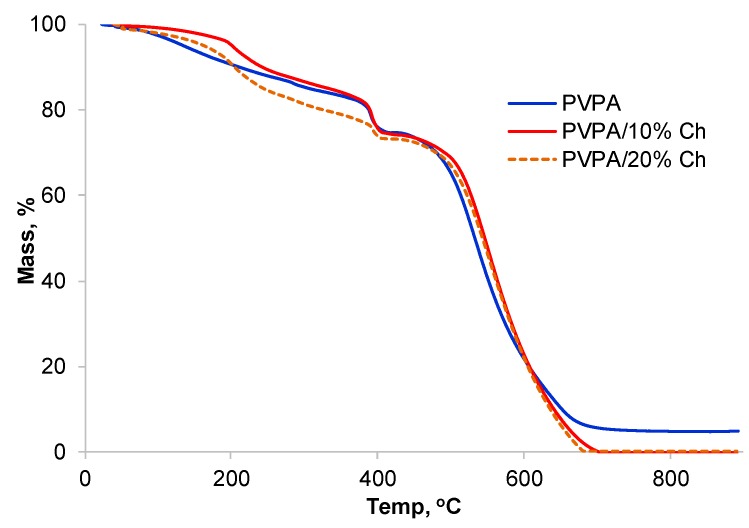
TGA curves for PVPA and chitosan containing plaques under air, with a heating rate of 10 °C per minute from RT to 900 °C.

Thermogravimetric analysis (TGA) (Figure 12) analysis showed that the presence of chitosan in PVPA has only a marginal effect on the first and second stages of mass loss, indicating that it should not interfere with the intumescence stage. In the third stage also there is no effect of chitosan on the mass loss rate but above 600 °C there is more mass loss from PVPA containing chitosan than from PVPA alone leading to no char residue. This is due to the complete decomposition of the chitosan component. These results are reflected in cone calorimetric results as well, with lower char thicknesses observed for PVPA coatings containing chitosan.

**Figure 13:**
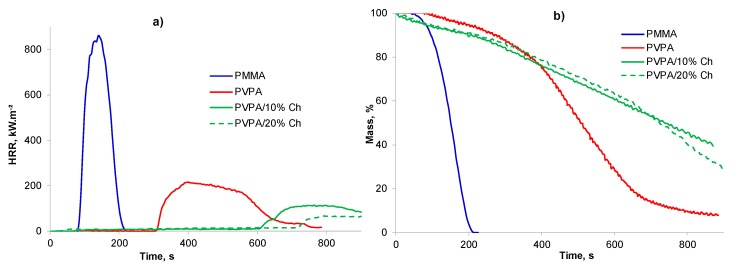
Plots of (**a**) heat release rate (HRR) vs. time and (**b**) mass loss vs. time for uncoated PMMA, PMMA coated with PVPA, and PMMA coated with PVPA/chitosan.

It can be seen from Table 2 and Figure 13 that in cone calorimetric tests, coatings on PMMA of crosslinked PVPA containing 10 wt% and 20 wt% added chitosan perform better than coatings of crosslinked PVPA alone (despite giving char heights lower than those produced by PVPA), with longer times to ignition, lower peak heat release rates, and lower total heat release.

**Figure 14:**
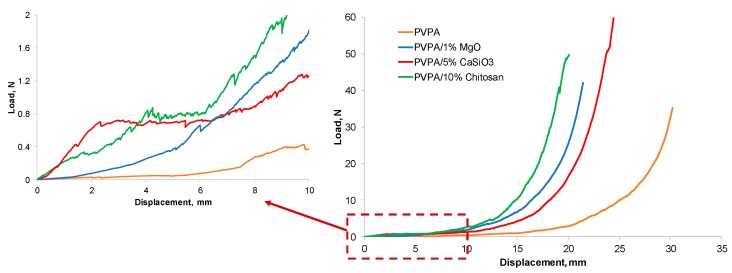
Load/displacement plots for chars from various PVPA based coatings.

The mechanical strengths of the chars after cone experiments, measured by a compressive test, are compared in Figure 14, where it can be seen that all PVPA chars are very soft initially with some level of elasticity. On applying load, the chars of all samples could resist the load to a limited extent and then the frail char collapsed slowly without showing clear fracture. After the initial elastic region, charred materials show densification behavior similar to those observed in cellular structures [19]. In the inset figure the elastic region has been expanded, where differences in behaviors of different chars can be clearly seen. PVPA char shows negligible stiffness, PVPA/1% MgO shows some ability to endure some load, but still no load vs. displacement plateau can be seen. PVPA/5% CaSiO_3_ gives the best results in terms of stiffness retention, in that it can withstand some load and shows plastic behavior (shown by the load vs. displacement plateau) before densification. This is expected as CaSiO_3_ forms a glassy structure on heating, which helps in maintaining this plastic region. The char from PVPA/10% chitosan also shows good stiffness retention, though not as pronounced as that from the CaSiO_3_ containing sample.

## 3. Discussion

Of the modifications to PVPA coatings on PMMA studied here, those containing added chitosan give the greatest improvement over PVPA alone with regards to water-tolerance (although dry adhesion to PMMA is slightly compromised), and have a fire-protective effect comparable with that PVPA. In fact, it can be argued that the fire protective effect of a PVPA/10% chitosan coating is better than that of PVPA alone in that it is able to prevent ignition of the underlying PMMA substrate at a lower coating thickness (0.27 mm vs. 0.42 mm) and that when ignition does occur, the time to ignition is longer, and both the peak heat release rate and the total heat released are lower, despite PVPA/10% chitosan forming a thinner, less intumescent, char layer than PVPA. It seems likely that at around the 10 wt% level, chitosan has a synergistic effect on the fire retardance of a PVPA coating, arising from acid-base pairing (hydrogen bonding) between -NH_2_ groups in chitosan and the -P-OH groups in PVPA. However at 20 wt%, chitosan is less effective in combination with PVPA (although still more effective than PVPA alone at similar thicknesses), possibly owing to some phase separation of the two components, such that much of the PVPA and chitosan are then burning essentially independently.

It is interesting to compare the fire protective performance of a PVPA/chitosan coating on a PMMA substrate with the performance of the best VPA copolymer coating reported in a previous paper [8], i.e., a coating based on the copolymerization of VPA with acrylonitrile (AN) (see Table 3).

It can be seen from the data in Table 3, that the VPA/chitosan coating outperforms the VPA/AN coating in every respect (wet and dry adhesion, and fire performance) except for THR, despite giving a lesser degree of intumescence. We conclude from this that the synergistic effect on fire retardance of nitrogen in the NH_2_ groups of chitosan is greater than the effect of nitrogen in the C≡N groups of acrylonitrile, because the latter does not form hydrogen bonds with PVPA. The relative fire performances of coated PMMA plaques can also be judged by comparing their fire growth rate (FIGRA) parameters [20], expressed in kW m^−2^ s^−1^, and calculated by dividing PHRR values by TTPHR values taken from Table 2 and Table 3 for those samples for which ignition occurred. These parameters are: No coating: 5.77; PVPA: 0.38, 0.48; P(VPA-co-AN): 0.56; PVPA/chitosan: 0.07. These values clearly indicate the superior fire performance of PVPA/chitosan coated PMMA plaques over the performances of the other samples.

## 4. Materials and Methods

### 4.1. Materials

Commercial poly(methyl methacrylate) (PMMA) sheet (Unpigmented, transparent, 3 mm thick, Lucite International UK Ltd., Southampton, UK) was used as received as the flammable polymer substrate to which PVPA-based coatings were applied.

Vinylphosphonic acid (VPA, BASF, Ludwigshafen, Germany), triallylisocyanurate (TAIC, TAICROS^®^, Evonik Performance Materials GmbH, Marl, Germany) and 2-hydroxy-2-methylpropiophenone (Darocur 1173, BASF, Ludwigshafen, Germany) were used as received as monomer, crosslinking agent and photoinitiator, respectively, for the preparation of crosslinked PVPA-based coatings.

As discussed previously, for one of the coating formulations containing chitosan, linear (non-crosslinked) PVPA was prepared by radical polymerization. The process used was based on a published method [21] with minor modifications and was as follows. VPA (50 g) was diluted with distilled water (12.5 mL), heated to 80 °C under nitrogen, and 63 mg of the initiator, 2,2′-azo-*bis*-isobutyronitrile (AIBN) added. After 3, 6 and 9 h, respectively, further amounts of AIBN (21 mg) and water (2.5 mL) were added to the reaction mixture on each occasion. After 12 h, heating was discontinued, and the solution allowed to cool. The aqueous solution of PVPA so obtained was used directly without isolation of the PVPA.

MgO, CaSiO_3_ and chitosan (Aldrich), used as additives in PVPA coatings, were used as received.

### 4.2. Methods

#### 4.2.1. PVPA Coatings and Discs

Crosslinked PVPA coatings were prepared on 75 mm × 75 mm plaques cut from PMMA sheet by first wrapping the plaques across the base and along the edges with aluminum foil to form a barrier within which to contain the initially fluid coating mixture. This barrier was secured to the plaque using a polymer clay support and nitrocellulose-based coating as a temporary adhesive and sealant. VPA (with additives in some experiments), crosslinking agent (TAIC, 10 wt% based on monomer weight) and photo initiator (HMP, 10 wt% based on monomer weight) were manually mixed with a glass rod. Approximately 3 g of coating was applied to the surface of each plaque by pouring followed by brushing to give an even coat generally between 0.25 and 0.5 mm thick. Following this, the coated samples were irradiated for 6 h in a photoreactor consisting of six 15 W UV black light bulbs, which have a peak emission at 360 nm wavelength. Irradiation distance was 30 mm. To eliminate the radical scavenging effect of oxygen during photo-polymerization, the photoreactor was purged with nitrogen (99.99% purity with a flow rate of 10 L/min) throughout the reaction. After UV irradiation, the polymer-coated PMMA sample was removed from the photoreactor and post-cured at 80 °C for 24 h in an oven. Major steps in the radical mechanism of photo-polymerization and crosslinking of VPA have been outlined in a previous publication [8].

Discs of solid coating materials, approximately 1.5 mm thick, were prepared by pouring 3 g of coating formulations into circular, 50 mm diameter, aluminum pans (Townson and Mercer) and photo-polymerizing as above. These samples, after removal from the pans, were used to assess the inherent solubility in water of cured coatings.

#### 4.2.2. PVPA/Inorganic Hybrid Coatings

Powdered MgO was dispersed in coating formulations by rapid stirring at loadings of 1, 5, and 10 wt%, so as to maintain a relatively low viscosity that allowed the formulation to be satisfactorily spread across the PMMA plaque or aluminum pan surface prior to photo-polymerization. Concentrations of MgO greater than 10 wt% gave very viscous mixtures.

CaSiO_3_ at 5 wt% loading was added to PVPA formulation during the mixing stage. With both MgO and CaSiO_3_, coating thicknesses were 0.4 ± 0.1 mm. For comparison PVPA coatings of similar thicknesses were prepared.

#### 4.2.3. PVPA/Chitosan Hybrid Coatings

Chitosan was introduced into PVPA coatings in two different ways:

(i) Sol-gel process: This process was adapted from that used by Cain et al. [17]. A 1 wt% aqueous solution of chitosan, to which a small amount of acetic acid had been added to aid solubility, was mixed with the aqueous solution of the pre-prepared linear PVPA (see Section 2.1) diluted to give a 2 wt% concentration of the polymer. On mixing the two solutions, a gel formed in which the PMMA substrate was soaked for 30 min before being removed and then dried in an oven at 80 °C for 3 h.

(ii) Chitosan addition: Chitosan was added at loadings of 10 wt% and 20 wt % to VPA monomer along with the TAIC and HMD prior to UV polymerization either on the PMMA substrate to form a coating or in an aluminum dish to give a disc of material.

Coating thicknesses were 0.25 ± 0.02 mm and 0.47 ± 0.02 mm with 10 and 20 wt% chitosan, respectively; PVPA coatings without chitosan of similar thicknesses were prepared for comparison purposes.

### 4.3. Characterization of Coatings

All substrate plaques were weighed before and after application of a coating and the wt% polymer deposited on the surface was calculated. The thicknesses of coatings were obtained from the difference between the thicknesses of coated and uncoated samples, measured using a digital caliper. It is assumed that the compositions of the coatings reflect those of the mixtures from which they are made and that, owing to the incorporation of a crosslinker, the molecular weights of the PVPA in the coatings are extremely high. It is thus not possible to confirm compositions nor molecular weights of the coatings by any solution-based techniques, e.g., solution-state NMR spectroscopy and GPC.

The flammabilities of PMMA plaques, with and without surface coatings, were evaluated using a cone calorimeter (Fire Testing technology, East Grinstead, UK) on samples measuring 75 mm × 75 mm; triplicate tests were carried out and the results averaged. The size of specimens used in this study is smaller than the standard size as dictated by ISO 5660-1 [22] owing to the limitation in the quantities of samples. We have discussed the effect of geometry dependence on fire performance in our previous publication [23], according to which there is not much effect on some key parameters, such as time to ignition (TTI) and peak heat release rate (PHRR). Moreover, the cone calorimeter data reported in this study are presented on a relative basis with respect to those of control samples. All samples were tested by exposing them to a 35 kW m^−2^ heat flux in the horizontal mode at a distance of 25 mm from the cone heater without a spark ignition source. The different flammability parameters are reported as time-to-ignition (TTI), time-to-flame out (TTFO), peak heat release rate (PHRR), time to PHRR (TTPHR), and total heat release (THR). The heights (thicknesses) of any char layers formed during the cone calorimeter tests (CH) are also reported.

Mechanical compressive strengths of the char residues remaining after the cone experiments were tested using the Instron 3369 Universal Testing System in a compressive mode with a 1 kN load cell at a cross head speed of 5 mm min^−1^. The samples were left to condition at room temperature prior to testing. A flat aluminum plate (75 mm × 75 mm), connected to the shaft of the load cell was used to cover the cross section of the sample during the testing process. The gauge length varied from 20 to 30 mm between samples depending on the intumescence of a particular coating. The load-displacement data were collected and analyzed using the Bluehill 3 software (Instron, High Wycombe, UK).

Tape pull tests were performed to evaluate the adhesion between a coating and the PMMA substrate, similar to the one specified in BS EN ISO 2409:2007 [24] often used to examine the adhesion of films or sheets to an adhesive surface. In this work a piece of Sellotape (25 mm × 50 mm) was applied on the surface of the coated sample (75 mm × 75 mm) and smoothed with fingers to ensure good contact. Holding the sample with one hand, the tape was then peeled back at an angle of 180° in one smooth movement with the other hand. The test was repeated three times on different locations on the same sample.

To evaluate the effect of water on coatings, coated PMMA plaques were subjected to a water-soak test, according to the BS EN ISO 2812-2:2007 standard [25]. The four edges of the coated plaque (35 mm × 35 mm specimen), with and without surface coatings, were sealed by applying epoxy resin before testing. After that the samples were fully immersed in 100 mL of deionized water at RT and removed after 24 h. Finally, the samples were dried at RT for 24 h and then at 100 °C for 2 h. Before and after the tape-pull test and the water-soak test, the samples were weighed to assess loss of material following the tests (MT and MS, respectively). The inherent solubilities in water of some coating formulations were also measured by subjecting small discs of the coating formulations to similar water-soak test, collecting undissolved residues by filtration, and then drying and weighing them to assess the amounts that had dissolved (MD).

## 5. Conclusions

This work has shown that PVPA-based organo-inorganic hybrid coatings can be prepared by complexing PVPA with different inorganic and organic species, the latter having minimal detrimental effect on the intumescence and fire-retardant barrier properties of these coatings. While both inorganic species (MgO and CaSiO_3_) improved the adhesion of the coating to a PMMA substrate during water-soak test, the best results were shown by organic species, chitosan. However, with all inclusions dry adhesion to PMMA was slightly compromised. Incorporation of these species, and in particular chitosan, in PVPA coatings on a typical thermoplastic has allowed us to achieve, we believe, a satisfactory balance between fire performance, dry adhesion, wet adhesion and water-solubility, such that these coatings could be used commercially in situations where a degree of resistance to water (e.g., use in a humid atmosphere) is required. However, there is still room for improvement, especially with regard to water-tolerance. Our research on these and similar coatings continues.

## Figures and Tables

**Table 1:** Results of water-soak and water-solubility tests on coatings and discs.

Sample *	CT/mm	MT/wt%	MS/wt%	MD/wt%
PVPA	0.42 ± 0.12	0.04	89	16
PVPA/1% MgO	0.40 ± 0.03	0.19	54	18
PVPA/5% MgO	0.53	0.34	52	19
PVPA/10% MgO	0.67	0.09	40	23
PVPA/5% CaSiO_3_	0.63 ± 0.04	0.17	97	15
PVPA ^ƚ^/chitosan (gel)	-	96	-	37
PVPA/10% chitosan	0.26 ± 0.01	0.67	24	16
PVPA/20% chitosan	0.48 ± 0.02	0.25	30	29

CT = Coating thickness, MT = mass lost from coating in tape-pull test, MS = mass lost from coating in water-soak test, MD = mass of disc dissolved in water solubility test. * Discs were all ca. 1.5 mm thick. ^ƚ^ This experiment used pre-prepared linear PVPA (see Experimental section).

**Table 2:** Results of cone calorimetry on a PMMA plaque coated with PVPA/MgO (1 wt%).

Coating on PMMA *	CT/mm	TTI/s	TTFO/s	PHRR/kW m^−2^	TTPHR/s	THR/MJ m^−2^	CH/mm
None	-	89 ± 3	275 ± 37	900 ± 44	150 ± 5	78 ± 1	-
PVPA	0.28 ± 0.02	338 ± 62	810 ± 20	190 ± 10	450 ± 70	60 ± 5	21 ± 1
	0.42 ± 0.9	No ignition					32 ± 6
		321	774	205	445	63	22
PVPA/1% MgO	0.37	No ignition					27
	0.44	723	1028	257	765	38	36
PVPA/5% MgO	0.53	33.7	378	659	343	425	62.3
PVPA/5% CaSiO_3_	0.60 ± 0.04	460 ± 170	1150 ± 40	154 ± 15	535 ± 185	48 ± 28	28 ± 3
PVPA/10% Ch	0.27	No ignition					13
	0.25	600	1490	115	765	44	16
PVPA/20% Ch	0.50	730	2015	70	1000	64	13
	0.47	390	1510	66	700	47	15

CT = coating thickness, TTI = time to ignition, TTFO = time to flame out, PHRR = peak heat release rate, TTPHR = time to peak heat release, THR = total heat released, CH = char height. * 75 mm × 75 mm plaque of PMMA.

**Table 3:** Tape-pull test, water-soak test and cone calorimetric data for PMMA substrates with crosslinked VPA/chitosan and VPA/AN coatings.

Sample *	CT/mm	MT/wt%	MS/wt%	TTI/s	TTFO/s	PHRR/kW m^−2^	TTPHR/s	THR/MJ m^−2^	CH/mm
PVPA/chitosan	0.5	0.25	30	730	2015	70	1000	64	13
P(VPA-co-AN)	0.5	0.9	47	385	675	247	440	43	28

* Both samples contained 20 wt% of the respective additive/comonomer (chitosan or AN).
